# Ganetespib with Methotrexate Acts Synergistically to Impede NF-κB/p65 Signaling in Human Lung Cancer A549 Cells

**DOI:** 10.3390/ph16020230

**Published:** 2023-02-02

**Authors:** Gehad Subaiea, Syed Mohd Danish Rizvi, Hemant Kumar Singh Yadav, Turki Al Hagbani, Marwa Helmy Abdallah, El-Sayed Khafagy, Hosahalli Veerabhadrappa Gangadharappa, Talib Hussain, Amr Selim Abu Lila

**Affiliations:** 1 Department of Pharmacology and Toxicology, College of Pharmacy, University of Ha’il, Ha’il 81442, Saudi Arabia; 2 Department of Pharmaceutics, College of Pharmacy, University of Ha’il, Ha’il 81442, Saudi Arabia; 3 Department of Pharmaceutics, School of Pharmacy, Suresh Gyan Vihar University, Jaipur 302017, India; 4 Department of Pharmaceutics and Industrial Pharmacy, Faculty of Pharmacy, Zagazig University, Zagazig 44519, Egypt; 5 Department of Pharmaceutics, College of Pharmacy, Prince Sattam Bin Abdulaziz University, Al-kharj 11942, Saudi Arabia; 6 Department of Pharmaceutics and Industrial Pharmacy, Faculty of Pharmacy, Suez Canal University, Ismailia 41522, Egypt; 7 Department of Pharmaceutics, JSS College of Pharmacy, JSS Academy of Higher Education and Research, Mysuru 570015, India

**Keywords:** anticancer, ganetespib, lung cancer, methotrexate, NF-κB/p65 signaling, NSCLC

## Abstract

Among the various types of cancer, lung cancer accounts for the highest number of fatalities across the globe. A combination of different cancer chemotherapeutics is regarded as an effective strategy for clinical management of different cancers. Ganetespib (GAN) is a well-established hsp90 inhibitor with enhanced pharmacological properties in comparison with its first-generation counterparts. Previous preclinical studies have shown that GAN exerts significant effects against cancer cells; however, its therapeutic effects against non-small cell lung cancer (NSCLC) A549 cells, achieved by modulating the expression of the NF-κB/p65 signaling pathway, remains unexplored. In this study, the combinatorial effect of GAN and methotrexate (MTX) against lung carcinomas was investigated through both in silico and in vitro studies. A combinatorial treatment regimen of GAN/MTX exerted more significant cytotoxic effects (*p* < 0.001) against A549 cells than individual treatments. The GAN/MTX combination also instigated nuclear fragmentation followed by augmentation in intracellular ROS levels (*p* < 0.001). The elevated ROS in A549 cells upon exposure to GAN/MTX combinatorial regimen was concomitantly accompanied with a remarkable reduction in mitochondrial viability. In addition, it was observed that the GAN/MTX combination succeeded in elevating caspase-3 activity and downregulating the expression levels of anti-apoptotic mediators Bcl2 and survivin in NSCLC A549 cells. Most importantly, the GAN/MTX combinatorial regimen impeded the activation of the NF-kB/p65 signaling pathway via repression of the expression of E-cadherin and N-cadherin, which was confirmed by molecular docking studies. Collectively, these findings demonstrated the synergistic effect of the GAN/MTX combinatorial regimen in suppressing the growth of A549 cells by modulating the NF-κB/p65 signaling pathway.

## 1. Introduction

With globally high incidence rates, lung cancer is reported to be a major cause behind cancer-associated deaths. In accordance with the latest reports of global cancer observation, it was estimated that 2,206,771 new cases of lung cases were reported, which contributed 11.4% of all the 19,292,789 cases of cancer during the year 2021. Among the reported cases of different types of cancers, lung cancer was responsible for 1,796,144 deaths, constituting 18% of a total of 9,958,133 deaths arising from various cancers globally [1]. Based on histopathological findings, lung cancer is differentiated into non-small cell lung cancer (NSCLC) and small cell lung cancer (SCLC). Moreover, it is now well-established that most of the diagnosed lung cancer cases fall under the characteristic attributes of NSCLC, whereas the remaining cases histopathologically resemble SCLC or are commonly of a mixed type [2]. During the 1970s, the initial chemotherapeutics involved in clinical management of lung carcinomas included doxorubicin and methotrexate; however, their clinical benefits were minimal [3]. With subsequent advancements, the clinical benefits of advanced chemotherapeutics, namely gemcitabine, vinorelbine, and taxanes were reported during the period 1980–1990 in the standard management of patients with lung carcinomas.

Currently, combination therapy for lung cancer treatment is one of the latest, most exciting advances in lung cancer research. It is believed that combinatorial treatment approaches constituted by different chemotherapeutics could result in significant benefits, particularly if the anticancer agents can act synergistically. Furthermore, the combination of chemotherapeutics overcomes the problem of clonal heterogeneity, which subsequently improves the response towards the treatment. Lastly, although combination therapy can be toxic, the toxicity might be significantly less if the two drugs in the combination work synergistically, which leads to a significant reduction in the drug doses used in the combination therapy, thereby alleviating the associated side effects [4]. 

The heat shock protein 90 (Hsp90) is an appealing target for cancer therapy due to its central role in oncogenic signaling. Many reports have demonstrated that high expression of Hsp90 is associated with tumor growth, and it is believed to be an oncogenic signaling node for cancer malignancy [5,6]. In addition, inhibiting Hsp90 activity has proven to disrupt multiple signal transduction pathways that are important for tumor development and survival [7]. GAN is an established inhibitor of Hsp90, which is structurally different from the prototypic ansamycin class, and it exhibits elevated biological and pharmacological attributes, especially in terms of safety and potency [8]. GAN was reported to exert potent antitumor activity in in vitro and in vivo models of NSCLC [9]. Furthermore, many reports have emphasized the potential of GAN, when used in a combinatorial approach, to elevate the clinical efficacy of standard chemotherapeutics in patients with various types of cancers [10,11,12]. Currently, several phase I-III clinical trials are now underway, with GAN being tested alone or in combination with other drugs to treat NSCLC, breast, ovarian, and other advanced adenocarcinomas [10,13,14,15]. 

NFκB is a critical signaling pathway that is extensively implicated in cancer development and progression. A mounting body of research has implied that chronic activation of NF-κB is related with the progression of several malignancies, such as breast, rectal and colon cancers [16,17]. Recently, several animal model and cell culture studies have demonstrated the links between NF-κB and lung carcinogenesis [18,19,20]. It was suggested that NF-κB in myeloid cells promotes lung cancer primarily through mediating inflammatory cytokine release in order to establish a cancer-prone inflammatory microenvironment [18]. Blocking the chronic activation of NF-κB was reported to instigate apoptotic cell death in cancerous cells along with eliciting concomitant decline in invasiveness and proliferation that further increases the sensitivity of cancerous cells towards anticancer therapeutics [21,22]. Majumdar et al. [23] have reported that MTX could efficiently inhibit the activation of NF-κB through the release of adenosine, which might contribute to the role of MTX in anti-inflammatory, immunomodulatory, and antiproliferative effects [23]. Furthermore, the capability of Hsp90 inhibitors to attenuate NF-κB-mediated transcription is the major basis for their anti-inflammatory properties. Inhibition of Hsp90 in most cell types leads to disruption in NF-κB signaling and prevents the nuclear translocation of NF-κB proteins [24]. These reports explicitly indicate the therapeutic potential of NF-κB in cancer management, including lung cancer. 

The aim of this study, therefore, was to explore the anticancer potential of the combination of GAN and MTX against NSCLC A549 cells. The results of this study emphasize that combining the Hsp90 inhibitor (GAN) with the purine biosynthesis inhibitor (MTX) synergistically augments the treatment’s cytotoxic potential against A549 by modulating the expression of NF-κB/p65 signaling. 

## 2. Results

### 2.1. Combination of GAN and MTX Exhibits Synergistic Effect against the Proliferation of Lung Cancer Cells

**Figure 1:**
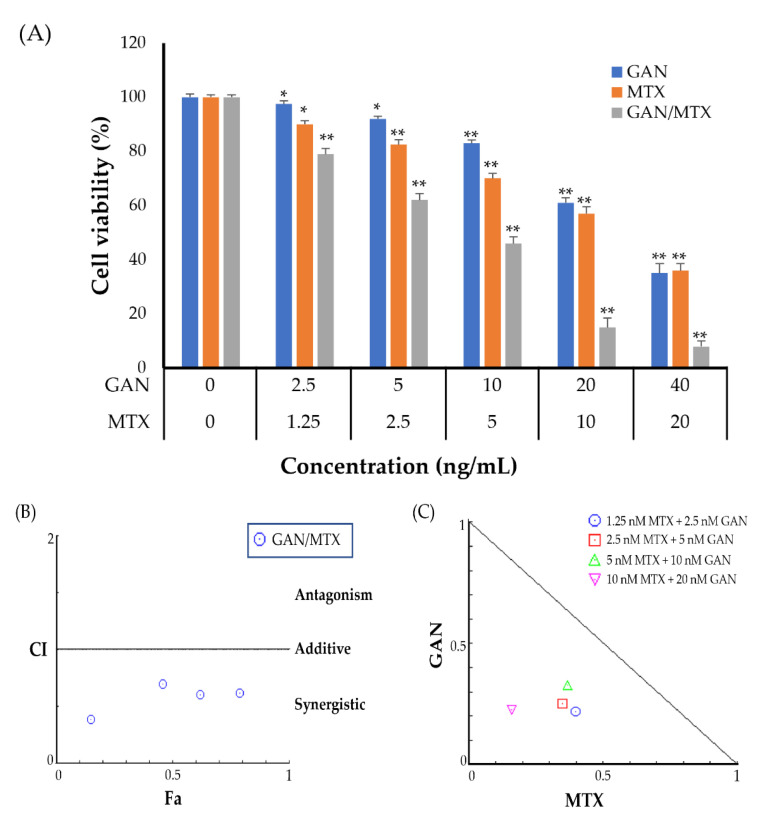
(**A**) Cell viability of A549 cells following treatment with either GAN or MTX alone or in combination. Data were expressed as mean ± SD. * *p* < 0.05 and ** *p* < 0.01 vs. untreated control. (**B**) Combination index plot (Fa-CI plot) of interaction between GAN and MTX against A549 cells. Fa: inhibitory effect, CI: combination index. An Fa of 0.5 represents 50% growth inhibition. (**C**) Isobologram generated with CompuSyn. Combination data points that fall below the line are synergistic; those above the line are antagonistic, and those on the line are additive.

We initially investigated the cytotoxic activity of GAN, MTX, and their combination on growth of A549 lung cancer cell lines using an MTT assay. As depicted in Figure 1A, monotherapy with GAN or MTX suppressed A549 cell viability in a dose-dependent manner compared to untreated control cells. In addition, the treatment of A549 cells with a combination of GAN and MTX elicited a further decline in cell viability compared to individual agents. The calculated IC_50_ value for GAN alone was 25 ± 3.27 nM, whereas that of MTX alone was 14 ± 2.42 nM. Based on the IC_50_ findings of monotherapy, a fixed constant ratio of MTX and GAN (1:2) was used to perform combination therapy with a combination regimen covering the IC_50_ values as well as higher and lower concentrations. Figure 1A shows that the combination of GAN/MTX inhibited A549 cell growth more potently than monotherapy and reduced the IC_50_ of GAN and MTX to 9.46 ± 3.56 nM and 2.79 nM ± 2.43 nM, respectively.

Furthermore, to address whether GAN could exert a synergistic effect with MTX on A549 lung cancer cells, the combination index (CI) was calculated by considering the half-maximal inhibitory concentration (IC_50_) data of the individual and combined cytotoxic effects of GAN and/or MTX. The calculated CI value was found to be 0.58, indicating a synergistic effect of combination therapy against A549 cells [25]. Figure 1B depicts the CI plot of the combination in A549 cells generated by CompuSyn software, which plotted CI against the inhibitory effect. The plot exposed a synergistic effect (CI < 1).

CompuSyn isobologram analysis (Figure 1C) confirmed our findings, and the data depicts the significant synergistic effect of GAN/MTX combination therapy against A549 cells at the doses between 1.25 to 10 nM of MTX and 2.5 to 20 nM for GAN, as indicated by point location under the slope. Collectively, our results suggest synergistic activity within the combinatorial therapy of GAN and MTX against A549 lung cancer cells.

### 2.2. Combination of GAN and MTX Exhibits Synergistic Effect in Augmenting ROS Production in Lung Cancer Cells

**Figure 2:**
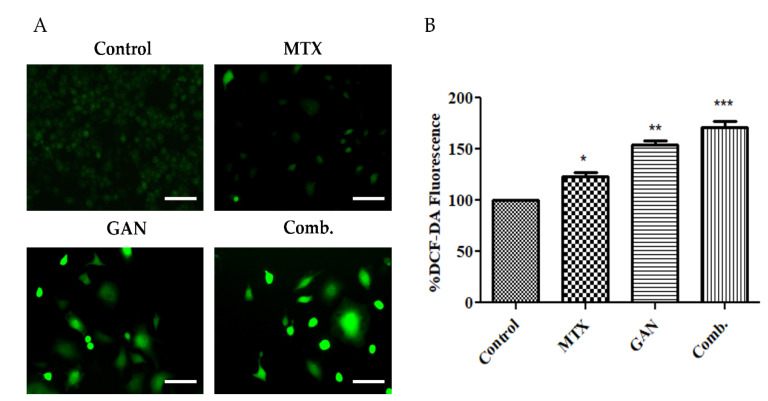
(**A**) ROS production in A549 cells with GAN and MTX alone or in combination compared to untreated control. (**B**) Quantification of augmented ROS in A549 cells after treatment with individual and combined doses of GAN and MTX compared to untreated control. Scale bar = 100 µm, Magnification: 20×. * *p* < 0.05, ** *p* < 0.01 and *** *p* < 0.001.

The treatment-mediated increased production of ROS with combined GAN and MTX was evaluated by staining with DCFH-DA dye. As shown in Figure 2, treatment with GAN and MTX combination therapy generated significantly higher levels of ROS in A549 cells compared to those observed in A549 cells treated with individual agents. These results suggest that the combined therapy with GAN and MTX synergistically augmented ROS generation in A549 cells.

### 2.3. Combination of GAN and MTX Exhibits Synergistic Effect in Inducing Nuclear Condensation and Fragmentation in Lung Cancer Cells

**Figure 3:**
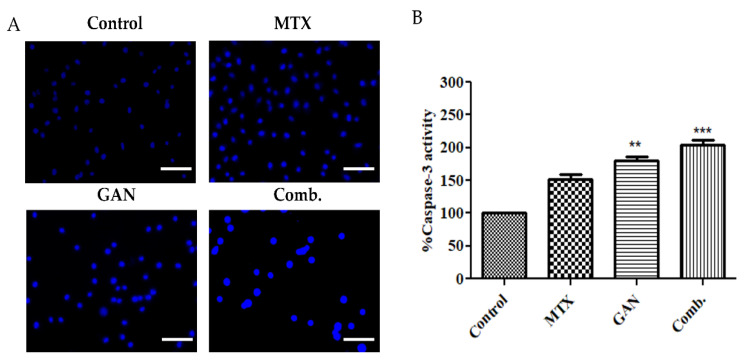
(**A**) Efficacy of individual and combined doses of GAN and MTX in inducing nuclear condensation and fragmentation in A549 cells compared to untreated control as analyzed with DAPI staining. Scale bar = 100 µm, Magnification: 20×. (**B**) Percent (%) caspase-3 activity in A549 cells after treatment with individual and combined GAN/MTX doses compared to untreated control. ** *p* < 0.01 and *** *p* < 0.001.

To investigate whether the treatment with GAN and MTX individually or in combination induced nuclear condensation and fragmentation in A549 cells, DAPI staining was performed. As shown in Figure 3A, the control cells did not show any nuclear condensation, which is indicated by diffused blue fluorescence. On the other hand, treatment with either GAN or MTX alone triggered remarkable nuclear condensation and fragmentation. Interestingly, A549 cells treated with a combination therapy of GAN and MTX demonstrated substantial nuclear condensation and fragmentation compared to their individual counterparts. These results suggest that GAN/MTX combination therapy exerts its cytotoxic potential, at least in part, via the induction of apoptosis in lung cancer cells.

### 2.4. Combination of GAN and MTX Induces Caspase Activation in Lung Cancer A549 Cells

Caspases are a family of protease enzymes playing essential roles in transmitting signals during programmed cell death (apoptosis). These enzymes, upon conversion from pro to active forms, mediate the proteolytic cleavage of various crucial cellular proteins. Accordingly, to investigate whether the synergistic apoptotic potential of the combination therapy of GAN and MTX against A549 cells was mediated via the activation of caspases, the activity of caspase-3, the major downstream effecter caspase in apoptotic cascade, was evaluated in A549 cells post-treatment with GAN and MTX individually and in combination. As shown in Figure 3B, treatment with GAN and MTX individually and in combination efficiently triggered a substantial induction of caspase-3 activity in A549 cells. Caspase-3 activity was considerably increased upon GAN/MTX combinatorial therapy compared to the individual doses of GAN and MTX (Figure 3B). 

### 2.5. Combination of GAN and MTX Exhibits Synergistic Effect in Dissipating Mitochondrial Membrane Potential in Lung Cancer Cells

**Figure 4:**
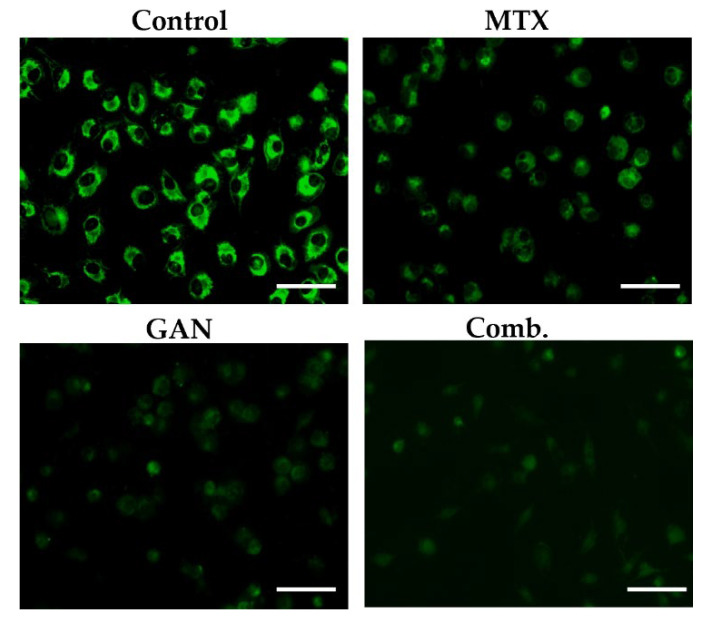
Dissipation of mitochondrial membrane potential in A549 lung cancer cells after treatment with individual and combined GAN/MTX doses compared to untreated control. Scale bar = 100 µm, Magnification: 20×.

Regulation of mitochondrial membrane potential plays an important role in cell survival. Loss of mitochondrial membrane potential triggers the release of cytochrome C and apoptosis inducing factor (AIF) from mitochondria to the cytoplasm, which is a potent activator of the apoptotic caspase cascade. Accordingly, dissipation of mitochondrial membrane potential is thought to be an early indication of apoptotic cell death [26]. To examine the impact of GAN and MTX alone or in combination on mitochondrial function, the mitochondrial membrane potential of A549 cells was measured at 12 h post-treatment using the mitochondrial voltage-specific reporter rhodamine 123 (Rh 123). As shown in Figure 4, untreated control A549 cells preserved an intact mitochondrial membrane potential, which is depicted by efficient Rh 123 dye cellular uptake resulting in a high fluorescence intensity. In contrast, treatment with either GAN or MTX exerted a negative impact on the integrity of the mitochondrial membrane as manifested by a remarkable decrease in Rh 123 dye-related fluorescence intensity. Most importantly, a combined treatment with GAN and MTX elicited a further decrease in the mitochondrial membrane potential in A549 cells compared to individual treatments with either GAN or MTX, validating the involvement of mitochondria-induced apoptosis in the cytotoxic effect of the combined therapy.

### 2.6. Combination of GAN and MTX Inhibits the NF-kB Signaling Pathway

**Figure 5:**
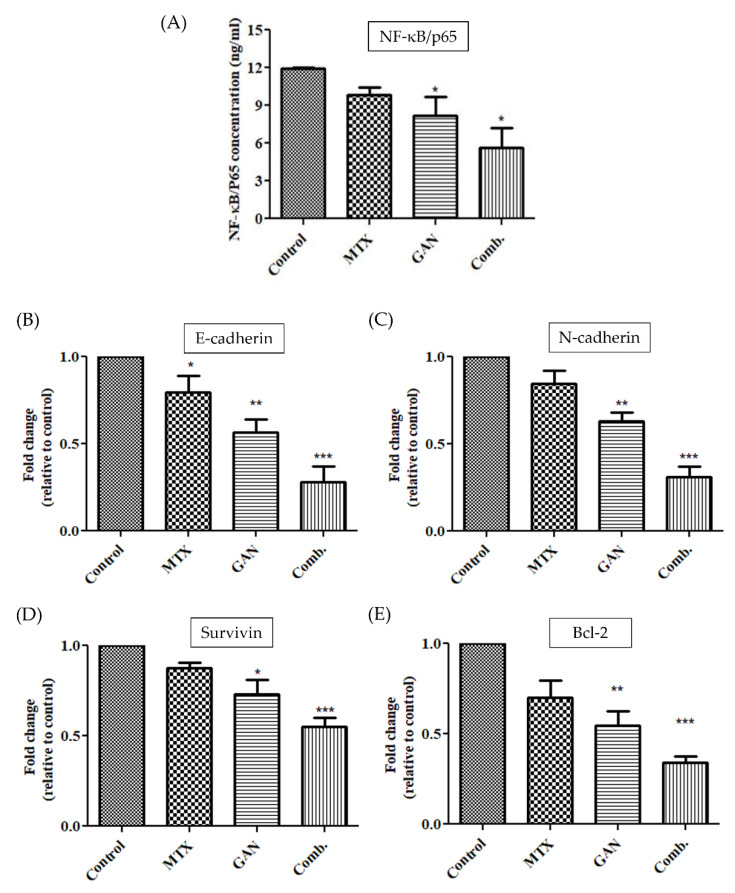
Effects of GAN and MTX alone or in combination on (**A**) concentration of NF-κB/p65; (**B**) mRNA expression of E-cadherin; (**C**) mRNA expression of N-cadherin; (**D**) mRNA expression of survivin; (**E**) mRNA expression of Bcl-2. * *p* < 0.05, ** *p* < 0.01 and *** *p* < 0.001.

A mounting body of reports have established that the NF-kB signaling pathway strongly contributes to the migration and invasion capabilities of cancer cells [27]. Accordingly, in order to obtain mechanistic insights into the combined treatment of GAN and MTX on A549 lung cancer cells, the levels of NF-kB/p65 were estimated using an Enzyme-Linked Immunosorbent Assay (ELISA). The results suggest that GAN and MTX in combination reduced the levels of NF-κB/p65 to 5.67 ± 1.26 ng/mL compared to their individual doses in A549 cells (Figure 5A). 

To gain insights into the mechanisms by which GAN and MTX combinatorial therapy mediates the downregulation of NF-κB/p65, the impact of combinatorial therapy on suppressing genes associated with the NF-kB signaling pathway, such as E-cadherin and N-cadherin, was investigated. As shown in Figure 5B,C, the mRNA expression level of both E-cadherin and N-cadherin were significantly downregulated after treatment with the synergistic combination of GAN and MTX compared with the drugs used individually. These findings validate that the synergistic combination of GAN and MTX can inhibit the migration and invasion of A549 cells via obstruction of the NF-kB signaling pathway.

It is well recognized that apoptosis is regulated by multiple factors. B-cell lymphoma 2 (Bcl-2) is a well-known anti-apoptotic mediator. Survivin is also a unique newly identified family inhibitor of apoptosis protein that controls apoptosis via a different pathway than Bcl-2 family members [28]. In this study, treatment with the GAN/MTX combination downregulated the mRNA expression of survivin and Bcl-2 to 0.55 and 0.43 folds, respectively, as compared to their individual treatment in lung cancer cells (Figure 5D,E). These findings support the concept that the synergistic combination therapy of GAN and MTX-induced apoptosis might be associated with not only the mitochondrial apoptotic pathway, which is particularly relevant to cancer, but also with the typical apoptotic signaling pathway as well.

### 2.7. Molecular Docking Results

**Figure 6:**
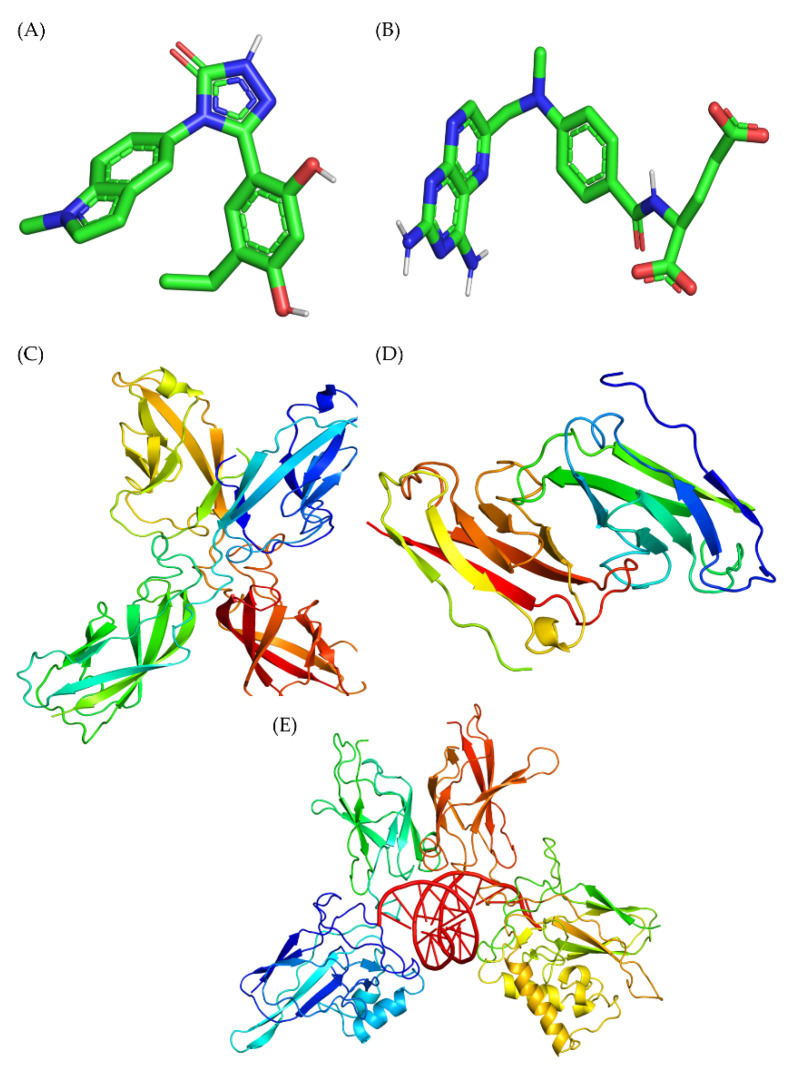
Three-dimensional chemical structures of (**A**) GAN, (**B**) MTX, (**C**) E-cadherin, (**D**) N-cadherin, and (**E**) NF-κB/p65.

**Figure 7:**
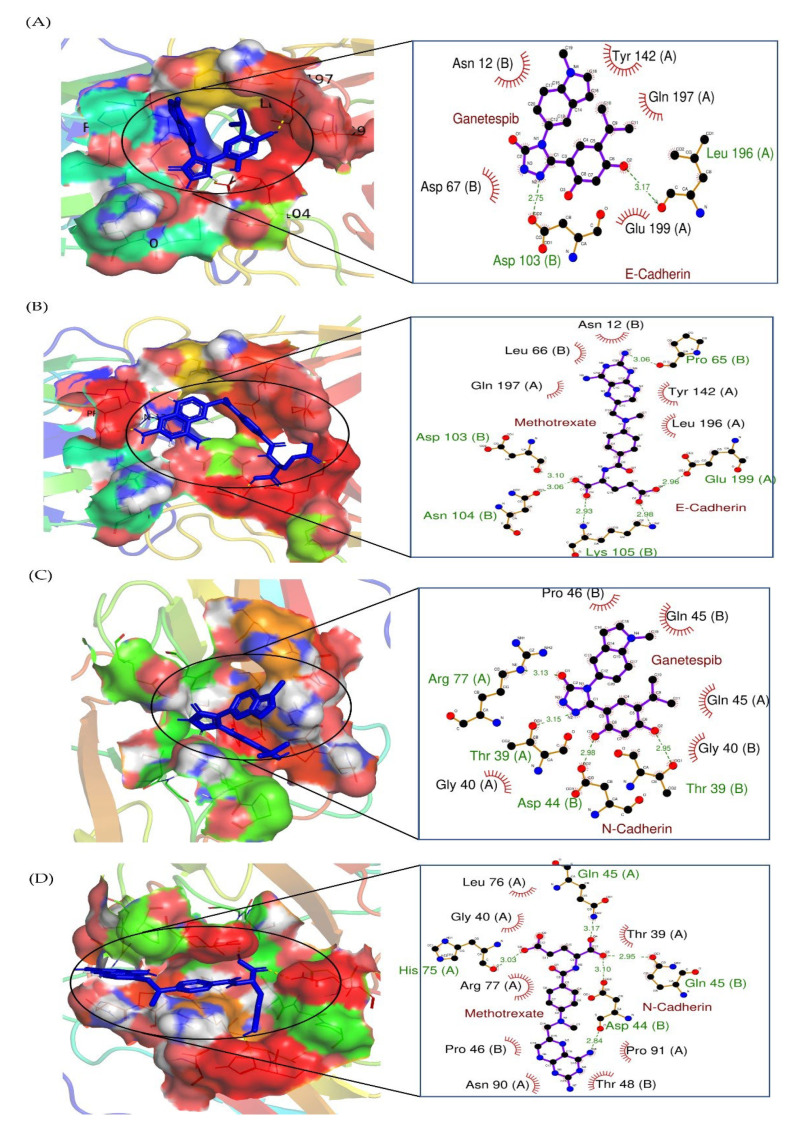
Two-dimensional interaction complex of (**A**) GAN and (**C**) MTX with E-cadherin protein, where red shows the target protein, purple shows the ligand molecule, and the black circle shows the enlarged view of docked complex. Two-dimensional interaction complex of (**B**) GAN and (**D**) MTX with N-cadherin protein, where red shows the target protein, purple shows the ligand molecule, and the black circle shows the enlarged view of the docked complex.

Molecular docking studies of GAN and MTX with E-cadherin, N-cadherin, and NFκB p65 were performed to predict the mechanism of lung cancer cell suppression, as shown in Figure 6. In the present study, our molecular docking investigations revealed that the binding energy of GAN towards E-cadherin and N-cadherin were −6.76 ± 0.02 and −6.23 ± 0.02 kcal/mol, respectively, which are nearer to the binding energies of MTX to E-cadherin and N-cadherin (−6.63 ± 0.02 and −6.56 ± 0.02 kcal/mol, respectively). Tyr 142 (A), Gln 197 (A), Glu 199 (A), Asp 67 (B), and Asn 12 (B) represent the residues that were involved in the hydrophobic interaction of GAN with E-cadherin. Leu 196 (A) (bond length 3.17) and Asp 103 (B) (bond length 2.75) residues were involved in hydrogen bonding (Figure 7A). In case of N-cadherin, Pro 46 (B), Gln 45 (B), Gln 45 (A), Gly 40 (B), and Gly 40 (A) residues were involved in hydrophobic interaction. Thr 39 (B) (bond length 2.95), Asp 44 (B) (bond length 2.98), Thr 39 (A) (bond length 3.15), and Arg 77 (A) (bond length 3.13) were involved in hydrogen bonding (Figure 7B). Contrastingly, the amino acids involved in the hydrophobic interactions with molecular docking of MTX with E-cadherin were Asn 12 (B), Tyr 142 (A), Leu 196 (A), Gln 197 (A), and Leu 66 (B) residues, whereas Glu 199 (A) (bond length 2.96), Lys 105 (B) (bond length 2.98 and 2.93), Asn 104 (B) (bond length 3.06), Asp 103 (B) (bond length 3.10), and Pro 65 (B) (bond length 3.06) amino acids were engaged in hydrogen bonding (Figure 7C). In case of N-cadherin, the interacting amino acid residues involved in hydrophobic interactions included Thr 39 (A), Pro 91 (A), Thr 48 (B), Asn 90 (A), Pro 46 (B), Arg 77 (A), Gly 40 (A), and Leu 76 (A) residues, whereas Gln 45 (A) (bond length 3.17), Gln 45 (B) (bond length 2.95), Asp 44 (B) (bond length 3.10 and 2.84), and His 75 (A) (bond length 3.03) residues were involved in hydrogen bonding (Figure 7D). The binding energies of GAN and MTX with lung cancer targets (E-cadherin and N-cadherin) and their interacting amino acids are summarized in Table 1.

**Figure 8:**
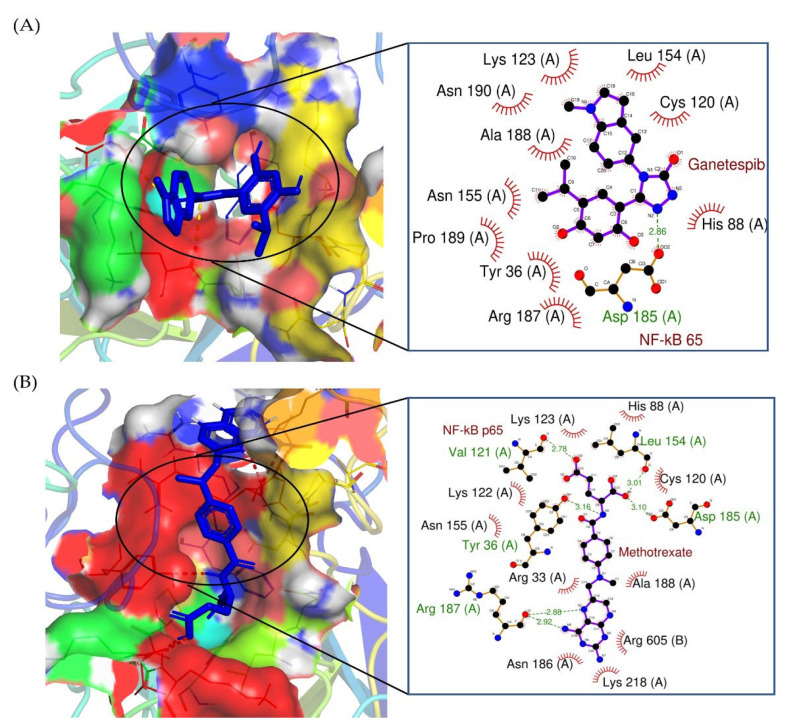
Two-dimensional interaction complex of (**A**) GAN and (**B**) MTX with NF-κB/p65 protein, where red shows the target protein, purple shows the ligand molecule, and the black circle shows the enlarged view of the docked complex.

Furthermore, molecular docking studies were performed to compare the binding energies of GAN-NF-κB/p65 and MTX- NF-κB/p65. It was found that the binding energy of the molecular docking of GAN with NF-κB/p65 was −6.36 ± 0.02 kcal/mol, and the amino acid residues Leu 154 (A), Cys 120 (A), His 88 (A), Arg 187 (A), Tyr 36 (A), Pro 189 (A), Asn 155 (A), Ala 188 (A), Asn 190 (A), and Lys 123 (A) were involved in hydrophobic interactions, whereas Asp 185 (A) (bond length 2.86) was engaged in hydrogen bonding (Figure 8A). However, during the molecular docking of methotrexate with NF-κB/p65 (−7.33 ± 0.05 kcal/mol), amino acid residues, namely His 88 (A), Cys 120 (A), Ala 188 (A), Arg 605 (B), Lys 218 (A), Asn 186 (A), Asn 155 (A), Lys 122 (A), and Lys 123 (A) were involved in hydrophobic interaction, whereas Leu 154 (A) (bond length 3.01), Asp 185 (A) (bond length 3.10), Arg 187 (A) (bond lengths 2.92 and 2.88), Tyr 36 (A) (bond length 3.16), and Val 121 (A) (bond length 2.78) were engaged in hydrogen bonding (Figure 8B). The binding energies of GAN and MTX with lung cancer targets (NF-κB/p65) and the interacting amino acids are summarized in Table 1. Thus, it can be concluded that both GAN and MTX are plausible inhibitors of the NF-κB signaling pathway in lung cancer cells.

## 3. Discussion

Despite the recent advancements in diagnosis and clinical management, elucidation of effective therapeutical modalities against NSCLC remains a formidable challenge for clinicians and oncologists globally. Combination therapy with conventional chemotherapeutic agents represents the frontline therapeutic strategy for the treatment of lung cancer [29]. Nevertheless, the major limitation of the currently used chemotherapeutics is the development of chemotherapy-induced toxicities, which lead to therapeutic dose reduction or delay and affects therapeutic patient outcomes. Instead, recent clinical trials have shown that the combination of targeted therapy with available standard chemotherapeutic agents could efficiently achieve greater therapeutic effects for treating lung cancer while minimizing the associated side effects [10,12]. In this study, we aimed to explore the synergistic anticancer effect of using the heat shock protein 90 (Hsp90) inhibitor, GAN, combined with the standard chemotherapeutic MTX against NSCLC in silico and in vitro. Our results explicitly show that GAN/MTX combination therapy synergistically suppressed the viability of A549 cancer cells compared to either GAN or MTX alone (Figure 1). Accordingly, our results revealed that the combination strategy of GAN/MTX is a promising one that might afford greater therapeutic effects compared to individual drugs.

GAN is reported to hold the potential of modulating the expression of several proteins involved in the functioning of cells, such as protein kinase B, cell cycle proteins, and Bcl-2 [9,11,30]. Previously, it was shown that the exposure of malignant cells to GAN impeded their growth and induced cellular apoptosis via the activation of the capsase-3/-7 cascade with concomitant reductions in the levels of total/phosphorylated AKT [31]. Furthermore, in models of NSCLC, GAN was reported to efficiently ameliorate several oncogenic factors and downregulate the expression of MAPK/AKT signaling to instigate apoptosis [32]. MTX, a folate antagonist, is a well-known cytotoxic agent which competitively and irreversibly inhibits dihydrofolate reductase, an enzyme that participates in tetrahydrofolate synthesis [33]. It is currently used in lung cancer treatment alone or along with other chemotherapeutic agents via oral and parenteral routes. In this study, we demonstrated that combination therapy with GAN/MTX adversely affects the integrity of the mitochondrial membrane and results in a remarkable dissipation of the mitochondrial membrane potential in lung cancer cells, which was more potent that that elicited by individual treatments with either GAN or MTX (Figure 4). In addition, combination therapy with GAN/MTX significantly downregulated the expression of antiapoptotic mediators (Bcl-2 and surviving) in A549 cells compared to individual treatments with either GAN or MTX (Figure 5), resulting in impaired mitochondrial outer membrane permeabilization and subsequently reduced mitochondrial membrane potential. Intriguingly, combination therapy with GAN/MTX substantially induced caspase activation in lung cancer A549. Collectively, these results imply that the combination therapy of GAN/MTX exerts its cytotoxic potential, at least in part, via several pathways, including instigating mitochondria-mediated apoptosis triggered by the dissipation of mitochondrial membrane potential, the downregulation of antiapoptotic mediators’ (Bcl-2 and survivin) expressions, and the activation of the caspase cascade (Figure 3).

Other than the loss of mitochondrial potential, excessive production of ROS is another important cellular event that plays a pivotal role in apoptosis induction under both physiologic and pathologic conditions [34]. It is well-recognized that the production of ROS is one of the possible events that can be triggered by the loss of mitochondrial integrity. Accordingly, in this study, the contribution of ROS generation to the cytotoxic potential of the combinatorial approach of GAN/MTX was elucidated. The treatment of A549 cells with the GAN/MTX combination resulted in remarkably higher intracellular ROS levels compared to individual treatments with either GAN or MTX (Figure 2), as evidenced by a substantial increase in the level of green fluorescence in cells treated with the combination therapy. These results confirmed that extensive ROS generation might contribute to the cytotoxic effect of combinatorial therapy of GAN/MTX against A549 cancer cells.

Nuclear factor-κB (NF-κB) is a transcription factor that plays a pivotal role in various biological processes, including inflammation, immune response, and cell growth and survival. Upregulation of NF-kB and its associated pathways was reported to promote cancer growth and progression by boosting angiogenesis and modifying the metabolic and immunological state within the tumor microenvironment [35,36]. In addition, in many clinical settings, the activation of NF-kB by chemotherapy and/or radiotherapy was associated with treatment failure and resistance [37]. Accordingly, therapeutic agents that target NF-kB might represent plausible strategies for cancer therapy. In this study, combination therapy with GAN/MTX efficiently decreased the expression of NF-κB/p65 (Figure 5A) and, hence, predicted ameliorations in the invasiveness and metastasis of A549 cells in in vivo model systems. Consequently, the expression levels of important genes related to metastasis, namely E-cadherin and N-cadherin, were investigated. E- and N-cadherin are calcium-binding proteins that assist in cell adhesion and play a crucial role in tumor metastasis [38,39,40,41]. Combination therapy with GAN/MTX efficiently downregulated the expression of both E- and N-cadherin (Figure 5B,C). Importantly, molecular docking studies validated the efficacy of combination therapy with GAN/MTX in impeding NF-κB/p65 signaling in A549 cancer cells. GAN and MTX both showed strong molecular interactions with calcium bind domain of E-cadherin (Asp103, Asn104 and Asp67). In addition, MTX exhibited strong interactions with His75 of N-cadherin, which is among the three important histidine residues (His75, His79 and His 110) involved in protonation-linked pH variations [42]. Furthermore, GAN and MTX showed strong interactions with the Tyr36, Leu154, Asp185, and Arg187 amino acid residues of NF-κB p65. Thus, in silico findings suggest that GAN and MTX could bind with cancer targets (cadherins and NF-κB) with equal efficacy. Cumulatively, these findings suggest that the combination of GAN and MTX inhibits lung cancer cells’ migratory and invasive capabilities, probably via suppression of the NF-κB signaling pathway.

## 4. Materials and Methods

### 4.1. Materials

3-(4,5-dimethylthiazol-2-yl)-2,5-diphenyl-2H-tetrazolium bromide (MTT) dye, rhodamine (Rh)123, and 2′, 7′-Dichlorofluorescin diacetate (DCF-DA) were commercially obtained from Himedia (Maharashtra, India). Dulbecco’s Modified Eagle Medium (DMEM), antibiotic–antimycotic solution, and fetal bovine serum (FBS) were commercially obtained from Gibco (Gaithersburg, MD, USA). The Caspase-3 Colorimetric Assay Kit used was purchased from BioVision (Waltham, MA, USA), and a Human NF-κB ELISA Kit (ab176648) was purchased from Abcam (Cambridge, UK). 

### 4.2. Cell Culture Maintenance

NSCLC A549 cells of human origin were obtained from the cell repository of the National Center of Cell Science (NCCS), Pune, India. The cells were allowed to proliferate in Dulbecco’s Modified Eagle Medium (DMEM) supplemented with fetal bovine serum (FBS) (10% *v*/*v*) and antibiotic-antimycotic solution (1%; *v*/*v*) in a humidified environment with CO_2_ levels of 5% at 37 °C. The cells were routinely monitored, and they were passaged after <90% confluency was attained. Cells were seeded in media supplemented with 10% FBS prior to experimentation after counting through a hemocytometer, as per the standard protocol.

### 4.3. Molecular Docking Studies

#### 4.3.1. Preparation of Ligands’ 3D Structure

The ligands of interest were selected on the basis of published literature and then further selected for molecular docking studies. The three-dimensional structures of the selected ligands, i.e., GAN [PubChem ID: 135564985] and MTX [PubChem ID: 126941], were retrieved from the PubChem database (www.pubchem.com, accessed on 9 September 2022) in SDF file formats. The SDF files of the desired ligands were converted into PDB formats for using AutoDock Vina 4.0 [43]. We utilized the Linux subsystem command line and used MMFF94 force field for energy minimization (EM); after EM, we prepared the ligand molecules by using mgltools 1.5.6.

#### 4.3.2. Retrieval of Targets’ 3D Structure

The three-dimensional crystal structures of target proteins such as E-cadherin (PDB ID 4ZTE), N-cadherin (PDB ID 1NCH), and NF-κB/p65 (PDB ID 1VKX) were utilized for performing molecular docking with ligands GAN and MTX. These three-dimensional structures were downloaded from the Protein Data Bank (www.rcsb.org/pdb; accessed on 9 September 2022). The structures of E-cadherin, N-cadherin, and NF-κB/p65 transcription factors employed for docking were devoid of any heteroatoms, including non-receptor atoms, namely water, ions, etc.

#### 4.3.3. Visualization of Docked Complex

All of the drug molecules were docked to the catalytic triad of proteins by using Autodock Vina 4, which was subsequently saved in a PDBQT file format. The population of potential ligand conformations/orientations at the binding site was estimated via docking. The binding pocket coordinates were chosen based on previously published literature, and the grid box was placed within a cubic box of magnitudes 40 × 40 × 40 [43]. The best-docked position with the lowest docked energy was chosen from nine conformations depending on the interacting residues, such as hydrogen bonds with a high binding affinity (kcal/mol). Using LigPlot, the protein–ligand interaction of docked complexes was shown in two dimensions [44]. All of the figures were generated with PyMol. Furthermore, Discovery Studio (https://discover.3ds.com/d, accessed on 21 September 2022) was utilized to study interactions between proteins and ligands in the PDB complex preparations. Eventually, a negative score (kcal/mol) was used to calculate the ligand’s binding energy affinity.

### 4.4. Cytotoxicity Assessment

The cytotoxic effects of GAN and MTX against NSCLC A549 cells was quantified either alone or with a combinatorial approach using MTT dye as described previously with slight alterations [45]. Initially, 1 × 10^4^ A549 cells were exposed to serial dilutions of either GAN or MTX for 24 h under optimum culture conditions. Subsequently, the treated and/or untreated cells were exposed to MTT dye (5 mg/mL) for another 4 h. After incubation, the cells were exposed to tissue culture grade DMSO (100 µL), and the plate was gently shaken for 30 min at room temperature in the dark. Eventually, the dissolved formazan was read for its absorbance at 570 nm using a spectrophotometer (Bio-Rad, CA, USA). The cytotoxicity of GAN and MTX was expressed as the percentage of cell viability with the following formula:

$$Cell\;Viability\;\left(\%\right)=\frac{Absorbance\;of\;treated\;NSCLC\;A549\;cells}{Absorbance\;of\;untreated\;NSCLC\;A549\;cells}\times 100$$

After IC_50_ determination, cells were treated with a combination of MTX and GAN at a constant ratio (1:2) in triplicate. The doses used in combination treatment were the IC_50_ as well as doses that were greater and lower than the IC_50_ of monotherapy [46]. The interaction efficacy of GAN, MTX, and their combination against A549 cells was estimated by calculating the combination index (CI) on the basis of results obtained from MTT by using the following formula:

Combination index (CI) = (D)_1_/(D_x_)_1_ + (D)_2_/(D_x_)_2_

where (D_x_)_1_ and (D_x_)_2_ are the doses (or concentrations) for D_1_ (GAN) and D_2_ (MTX) alone that give x % inhibition, whereas (D)_1_ and (D)_2_ are the doses of GAN and MTX in combination that also inhibit x %. CI < 1, CI = 1, and CI > 1 indicate synergism, additive effects, and antagonism, respectively [25].

CompuSyn software (Version 1.0; Combosyn, Paramus, NJ, USA) was also adopted to conduct isobologram analysis and determine synergism.

### 4.5. Morphological Assessment of A549 Cells

The effect of GAN and MTX on the morphology of A549 cells alone and in combination was also evaluated using a bright field microscope. The protocol was nearly identical to that of MTT as discussed above. Briefly, 1 × 10^4^ A549 cells were exposed to stated concentrations of GAN and MTX either alone or in combination. The treated and untreated control cells were incubated under ambient culture conditions for 24 h. Thereafter, the cells of different groups were visualized for changes in the morphological features of A549 cells, and images were captured using a FLoid Imaging Station, Thermo-Scientific (Waltham, MA, USA). Comparisons in morphological attributes were made using untreated control A549 cells.

### 4.6. Assessment of Nuclear Morphology

Changes within nuclear morphology resulting in the onset of apoptosis of A549 cells post-exposure with GAN, MTX, and their combination were assessed through Hoechst 33,342 dye [47]. 1 × 10^5^ cells were exposed to the stated concentration of GAN and MTX either alone or in combination for 24 h. Subsequently, the cells were washed with PBS and exposed to 5 µg/mL of Hoechst 33,342 dye for 10 min in ambient culture conditions. Finally, the treated cells were visualized and recorded using blue filters with a FLoid Imaging Station, Thermo-Scientific. The level of characteristic blue fluorescence in different groups was qualitatively compared with that of the untreated control A549 cells.

### 4.7. Caspase-3 Activity

The activity of caspase-3 was further investigated colorimetrically using a commercially available kit (BioVision, Waltham, MA, USA). 1 × 10^6^ A549 exposed to GAN, MTX, and their combination for 24 h were lysed on ice for 10 min using a chilled lysis buffer (50 mL). Thereafter, the supernatant was collected post-centrifugation (10,000× *g*; 1 min) and placed on ice. Subsequently, protein quantification was undertaken, and the samples were again re-diluted with chilled lysis buffer (50 mL). The lysate was then added to each well of a 96-well plate coated with DTT (10 mM) followed by supplementation with DEVD-pNA (4 mM), and the plate was left undisturbed for 1 h at 37 °C. Finally, the absorbance of each group was read and recorded at 405 nm. Results were interpolated as increases in activity percentages by comparing each group with the control group.

### 4.8. Evaluation of Intracellular ROS

ROS generation within the NSCLC A549 cells treated with GAN and MTX either alone or in a combinatorial approach was qualitatively and quantitatively assessed using the DCFH-DA stain as stated previously [48]. During the qualitative assessment of ROS, 1 × 10^4^ NSCLC A549 cells were exposed to ganetespib and methotrexate either alone or in combination for 12 h. Thereafter, each treated and/or untreated group was supplemented with DCFH-DA (10 µM; 100 µL) and incubated for an additional 30 min at 37 °C in the dark. Finally, A549 cells were washed once gently through 1× PBS and were visualized at an excitation/emission ratio of 482/18 nm: 532/59 nm under the green filter of a FLoid imaging station.

For quantitative assessment, 1 × 10^5^ A549 cells were seeded in each well of a 6-well plate and exposed to GAN and MTX either alone or in combination (at stated concentrations). Subsequently, the treated and/or untreated cells were pelleted (1500 rpm; 37 °C for 2 min), and the pellets were mixed gently with DCFH-DA (10 µM) and were allowed an additional 30 min of incubation at 37 °C in the dark. Finally, the cells belonging to different groups were read for their DCF-DA mediated fluorescence using a Synergy H1 Hybrid multi-mode microplate Reader (BioTek, Winooski, VT, USA). The results were expressed in terms of fluorescence intensity percentages compared with the untreated control A549 group.

### 4.9. Assessment of Mitochondrial Membrane Potential

Mitochondrial viability in A549 cells was further assessed through Rhodamine (Rh)-123 staining per the protocol described previously [49]. Briefly, 1 × 10^4^ A549 cells were exposed to stated concentrations of GAN and MTX either alone or in combination for 12 h. Subsequently, the cells were re-exposed to the Rh-123 stain (5 mg/mL) and incubated in the dark at room temperature for 30 min. Finally, the wells were decanted, and A549 cells belonging to various groups were visualized and recorded for their associated green fluorescence through a FLoid imaging station.

### 4.10. Assessment of NF-κB Levels

The effects of GAN and MTX alone and in combination were investigated to elucidate alterations to the NF-κB levels of A549 cells. The levels of NF-κB/p65 in treated and/or untreated NSCLC A549 cells were evaluated colorimetrically using a human-specific NF-κB/p65 ELISA kit (ab176648); Abcam, Cambridge, UK).

### 4.11. qRT-PCR Based Assessment of Gene Expression

1 × 10^6^ A549 cells were exposed to GAN, MTX, and their combination at the above stated concentrations for 24 h. Subsequently, total RNA content from A549 cells of different groups were extracted with an RNA Miniprep purification kit (Himedia, Pune, India). 2 µg of the RNA content from different groups was used for synthesizing cDNA using a Verso cDNA synthesis kit (Thermo-Fisher Scientific, Waltham, MA, USA). The primers used in the study are shown in Table 2. Lastly, qPCR was performed using a SYBR Green qPCR Kit by following the manufacturer’s protocol (Thermo-Fisher Scientific, Waltham, MA, USA). Normalization was done with a GAPDH gene, the data was analyzed using the comparative CT method, and the results were expressed in fold change as calculated using the 2ΔΔCT method.

### 4.12. Statistical Assessments

The data were expressed as mean ± SEM of three individual experiments performed at least thrice. The means of individual groups were compared with that of the control group by using one-way ANOVA subsequently followed by Dunnett’s multiple comparison post-hoc tests using GraphPad Prism (Ver. 5) software. Differences in the means of various groups were considered statistically significant when * *p* < 0.05, ** *p* < 0.01, and *** *p* < 0.001.

## 5. Conclusions

The results of the present study demonstrate that the combination of GAN and MTX had a synergistic effect against the growth and proliferation of lung cancer cells, resulting in relevant dose reduction of each drug used in the combination. The GAN/MTX combination substantially inhibited the growth of NSCLC A549 cells by instigating mitochondria-mediated apoptosis. In addition, the combinatorial use of GAN and MTX obstructed the migration and invasion of lung cancer cells by alleviating the activation of the NF-κB signaling pathway. Collectively, the combinational anticancer therapeutic effects of GAN/MTX might represent a viable therapeutic strategy for the treatment of NSCLC. Nevertheless, further in vivo studies are required for fully elucidating the efficacy of this combination therapy for NSCLC.

## Figures and Tables

**Table 1:** Binding energies of GAN and MTX with lung cancer targets (E-cadherin, N-cadherin, and NF-κB p65) and the interacting amino acids.

Compound	Binding Energy (kcal/mol)	Hydrogen Bonds	Hydrophobic Interactions	pKi (kcal/mol)
Ganetespib-E-cadherin	−6.76 ± 0.02	Leu 196 (A) (bond length 3.17) and Asp 103 (B) (bond length 2.75) residues were involved in hydrogen bonding.	Tyr ^142^ (A), Gln ^197^ (A), Glu ^199^ (A), Asp ^67^ (B), and Asn ^12^ (B)	−11.41
Methotrexate-E-cadherin	−6.63 ± 0.02	Glu ^199^ (A) (bond length 2.96), Lys ^105^ (B) (bond length 2.98 and 2.93), Asn ^104^ (B) (bond length 3.06), Asp ^103^ (B) (bond length 3.10), and Pro ^65^ (B) (bond length 3.06) amino acids engaged in hydrogen bonding.	Tyr ^142^ (A), Gln ^197^ (A), Glu ^199^ (A), Asp ^67^ (B), and Asn ^12^ (B)	−11.19
Gantespib-N-cadherin	−6.23 ± 0.02	Thr 39 (B) (bond length 2.95), Asp 44 (B) (bond length 2.98), Thr 39 (A) (bond length 3.15), and Arg 77 (A) (bond length 3.13) were involved in hydrogen bonding.	Pro 46 (B), Gln 45 (B), Gln 45 (A), Gly 40 (B), and Gly 40 (A)	−10.51
Methotrexate-N-cadherin	−6.56 ± 0.02	Gln 45 (A) (bond length 3.17), Gln 45 (B) (bond length 2.95), Asp 44 (B) (bond length 3.10 and 2.84), and His 75 (A) (bond length 3.03) residues were involved in hydrogen bonding.	Thr ^39^ (A), Pro ^91^ (A), Thr ^48^ (B), Asn ^90^ (A), Pro ^46^ (B), Arg ^77^ (A), Gly ^40^ (A), and Leu ^76^ (A)	−11.07
Gantespib-NF-κB p65	−6.36 ± 0.02	Asp 185 (A) (bond length 2.86) engaged in hydrogen bonding.	Leu 154 (A), Cys 120 (A), His 88 (A), Arg 187 (A), Tyr 36 (A), Pro 189 (A), Asn 155 (A), Ala 188 (A), Asn 190 (A), andLys 123 (A)	−10.73
Methotrexate-NF-κB p65	−7.33 ± 0.05	Leu ^154^ (A) (bond length 3.01), Asp ^185^ (A) (bond length 3.10), Arg ^187^ (A) (bond length 2.92 and 2.88), Tyr ^36^ (A) (bond length 3.16), and Val ^121^ (A) (bond length 2.78) engaged in hydrogen bonding.	His 88 (A), Cys 120 (A), Ala 188 (A), Arg 605 (B), Lys 218 (A), Asn 186 (A),Asn 155 (A), Lys 122 (A), and Lys 123 (A)	−12.37

**Table 2:** List of primers (forward and reverse) used in the present study.

Gene Name	Forward Primer	Reverse Primer
GAPDH	CGACCACTTTGTCAAGCTCA	CCCCTCTTCAAGGGGTCTAC
Bcl2	ATTGGGAAGTTTCAAATCAGC	TGCATTCTTGGACGAGGG
Survivin	ACCGCATCTCTACATTCAAG	CAAGTCTGGCTCGTTCTC
E-cadherin	CAGGTCTCCTCATGGCTTTGC	CTTCCGAAAAGAAGGCTGTCC
N-cadherin	TCACCAACTGGGACGACAT	CACAGCCTGGATAGCAACG

## Data Availability

Not applicable.
